# A prospective study of the influence of the skeleton on calcium mass transfer during hemodialysis

**DOI:** 10.1371/journal.pone.0198946

**Published:** 2018-07-30

**Authors:** Patricia Taschner Goldenstein, Fabiana Giorgeti Graciolli, Gisele Lins Antunes, Wagner Vasques Dominguez, Luciene Machado dos Reis, Sharon Moe, Rosilene Motta Elias, Vanda Jorgetti, Rosa Maria Affonso Moysés

**Affiliations:** 1 Nephrology Division, Universidade de São Paulo, São Paulo, Brazil; 2 Indiana University School of Medicine and Roudebush Veterans Administration Medical Center, Indianapolis, United States of America; 3 Medicine Master Degree Program, Universidade Nove de Julho (UNINOVE), São Paulo, Brazil; Universidade Estadual Paulista Julio de Mesquita Filho, BRAZIL

## Abstract

**Background:**

Calcium gradient, the difference between serum calcium and dialysate calcium d[Ca], is the main contributor factor influencing calcium transfer during hemodialysis. The impact, however, of bone turnover, on calcium mass transfer during hemodialysis is still uncertain.

**Methods:**

This prospective cross-sectional study included 10 patients on hemodialysis for a 57.6±16.8 months, with severe hyperparathyroidism. Patients were submitted to 3 hemodialysis sessions using d[Ca] of 1.25, 1.5 and 1.75 mmol/l in three situations: pre-parathyroidectomy (pre-PTX), during hungry bone (early post-PTX), and after stabilization of clinical status (late post-PTX). Biochemical analysis and calcium mass transfer were evaluated and serum bone-related proteins were quantified.

**Results:**

Calcium mass transfer varied widely among patients in each study phase with a median of -89.5, -76.8 and -3 mmol using d[Ca] 1.25 mmol/L, -106, -26.8 and 29.7 mmol using d[Ca] 1.50 mmol/L, and 12.8, -14.5 and 38 mmol using d[Ca] 1.75 mmol/L during pre-PTX, early post-PTX and late post-PTX, respectively, which was significantly different among d[Ca] (p = 0.0001) and among phases (p = 0.040). Ca gradient and delta of Ca also differed among d[Ca] and phases (p<0.05 for all comparisons), whether ultrafiltration was similar. Serum Osteocalcin decreased significantly in late post-PTX, whereas Sclerostin increased earlier, in early post-PTX.

**Conclusions:**

The skeleton plays a key role in Ca mass transfer during dialysis, either by determining pre-dialysis serum Ca or by controlling the exchangeable Ca pool. Knowing that could help us to decide which d[Ca] should be chosen in a given patient.

## Introduction

Disturbances in mineral and bone metabolism in chronic kidney disease patients (CKD-MBD) are highly prevalent and are a major cause of morbidity and mortality. Calcium (Ca) is an essential ion in the management of CKD-MBD. The serum and intracellular levels of Ca are critical in the maintenance of normal physiologic processes. Similarly, Ca balance (net intake minus output) in adults is important in ensuring no excess Ca load that may predispose to extracellular calcification. In patients with CKD, not yet on dialysis, formal balance studies suggest that positive Ca balance occurs at intake levels of 800 mg/day [1, 2]. Unfortunately, such studies cannot be done in patients on dialysis due to the Ca changes that occur acutely with dialysis. When patients reach the need for dialysis, Ca mass transfer from dialysate may alter the overall balance in order to extrapolate what we know about Ca balance from pre-dialysis patients to dialysis patients.

Currently, there is considerable controversy in the literature about the optimal Ca concentration in the dialysate (d[Ca]), and recommendations of a d[Ca] of 1.25, 1.5 or 1.75 mmol/L are mostly opinion-based. For those patients with suspected adynamic bone disease, some studies have shown benefits from using a d[Ca] of 1.25 mmol/L on bone and mineral parameters [3, 4]. Most nephrologists and current guidelines believe that an ideal d[Ca] should provide a near-neutral Ca balance during dialysis [5–7]. Yet, only limited studies have evaluated the quantity of Ca that moves between patient and dialysate [8–15]. This is mainly due to technical difficulties in obtaining an accurate measurement of Ca in the spent dialysate. Also, we must keep in mind that there are different pools of calcium in blood, the protein-bound, and the diffusible Ca, composed by the ionized Ca (iCa) and the Ca complexed with phosphate and citrate, which increases the difficulty to calculate the intradialytic Ca balance.

Multiple factors are involved in Ca mass transfer during a dialysis treatment. First, the gradient between d[Ca] and serum Ca plays a major role in this process. Second, charged particles such as proteins can interfere with the transfer of calcium from the blood to the dialysate, a process named Gibbs-Donnan effect [16]. Third, different treatments may alter Ca levels and/or Ca balance such as calcitriol, Ca based phosphate binders, and calcimimetics. Finally, the skeleton also seems to impact intra-dialytic Ca balance: there is an exchangeable Ca pool on the bone surface composed of non-collagenous proteins with high Ca affinity that could act as a reservoir for rapid exchanges with the extracellular Ca [17]. Our group has previously shown that Ca balance is dependent not only on Ca gradient, but also on bone turnover, as differences in mass transfer were observed in patients with high compared to low bone remodeling [8]. A limitation of this study was the cross-sectional design, which compared different patients at just one moment such that specific individual factors such as age, gender, mobility, and the severity of bone disease were not considered, and might have played a role in determining different Ca balances. Therefore, we designed a prospective study in dialysis patients with different states of bone turnover: pre and post PTX, where patients bone remodeling goes from high to low turnover. These patients were submitted to consecutive dialysis sessions with different d[Ca] before PTX, during hungry bone syndrome (early post-PTX), and after stabilization of bone disease (late post-PTX).

## Materials and methods

### a. Study design

This was a prospective cohort study, where patients were submitted to 3 dialysis sessions with different d[Ca] in each of the 3 consecutive phases of the study:

1. Pre PTX: SHPT while waiting for PTX (within 90 days pre PTX)
2. Early post-PTX: During the “hungry bone syndrome”, defined as the post operative period after PTX in which there is a severe hypocalcemia and hypophosphatemia with necessity of supplementation of Ca and calcitriol with elevated alkaline phosphatase. All procedures, in this phase, were done within 14 days of PTX, and always after weaning from IV Ca infusion.
3. Late post-PTX: after stabilization of bone remodeling, defined as no need for Ca supplements or normalization of serum alkaline phosphatase.

During each phase participants were submitted to 3 randomly assigned, consecutive bicarbonate-based hemodialysis sessions, with different d[Ca]: 1.25 mmol/L, 1.5 mmol/L and 1.75 mmol/L. Each session lasted 4 hours or a little longer to reach 240 min of effective dialysis time, with blood and dialysate flow of 350 and 800 ml/min, respectively. Ultrafiltration was adjusted according to each patient’ dry weight.

### b. Participants

Eighteen patients were included between July 2011 and July 2013. All subjects gave informed written consent to participate in the study, which was approved by our Institutional Review Board in accordance with the Declaration of Helsinki. Inclusion criteria were: age 18 or older, CKD patients on dialysis for more than 3 months who attended the CKD-MBD clinic at Hospital das Clínicas da Universidade de São Paulo and the presence of severe SHPT defined as PTH over 800 pg/mL with clinical indication for a PTX. All patients had the diagnosis of high bone remodeling confirmed by bone biopsy in the beginning of the study.

### c. Blood and dialysate measurements

Blood samples were collected on the arterial dialysis tubing before and every 30 min during each dialysis session for biochemical analysis, which included: total Ca (tCa), iCa, phosphate (P), urea, and intact PTH using routine laboratory techniques. Blood samples for measurement of bone-related proteins were collected pre-dialysis, on the first dialysis session of each of the three phases of the study. These samples were centrifuged, aliquoted in eppendorfs, and stored at -80° C. Serum proteins were then quantified through Multiplex Milliplex map kit–Human Bone Magnetic Bead Panel—HBNMAG-51K (EMD Millipore Corporation, MA, USA®) assay that quantified Dkk1, Leptin, FGF-23, sclerostin, osteoprotegerin and total osteocalcin (OC). Carboxylated OC (GLA) and undercarboxylated OC (GLU) fractions were measured through ELISA assay from Takara® (Japan).

Fresh dialysate samples were collected every 30 min to ensure the maintenance of Ca delivery in the dialysate. We used a partial spent dialysate collection method, which was shown to correlate very well with total spent dialysate collection [18]. Through this technique, spent dialysate and ultrafiltrate were continuously sampled by a reversed automatic injection pump, located in the waste tubing just before the drain, throughout the complete dialysis procedure at a rate of 1L/h as previously described [18–20]. This system ensured a constant volume of fluid ejecting it at a 5L capacity recipient. Samples of the pulled fluid were analyzed every 30 min for total Ca measurement. At the end of procedure all diverted fluid was homogenized and three samples were collected for calculation of Ca mass transfer.

### d. Ca mass transfer and ca gradient

Ca dialysate mass transfer (net amount of Ca put into or taken out of dialysate) was calculated using the formula: *Ca Mass Transfer* = [final dialysate volume (L)* final dialysate total Ca (mmol/L]–[dialysate volume (L)]* [pre capillary fresh dialysate Ca (mmol/L)]; where: final dialysate volume = dialysate volume (L) + ultrafiltrate volume (L); dialysate volume = 4h x 800ml/min = 192 L (it is fixed); ultrafiltrate volume = adjusted according to patient’s dry weight; final dialysate Ca = average of the three total Ca collected at the final homogenized diverted spent dialysate; pre capillary fresh dialysate total Ca = Ca measured at the fresh dialysate in the pre filter capillary.

Ca Gradient, which is the difference between blood and dialysate calcium concentrations, was calculated through the following formulas: *tCa Gradient* = total serum Ca pre dialysis (mmol/L)–initial pre capillary fresh dialysate Ca (mmol/L) and *iCa Gradient* = ionized serum Ca pre dialysis (mmol/L)–initial pre capillary fresh dialysate Ca (mmol/L).

### e. Statistical analysis

Continuous variables were expressed as mean ± standard deviation or median and percentiles (25; 75), according to the D'Agostino & Pearson omnibus normality test. Categorical variables were expressed as N and percentage. Analysis of variance (ANOVA) for repeated measures or alternative nonparametric Friedman test were used to compare variables in the three different d[Ca] in each phase of the study. ANOVA or the alternative Kruskall-Wallis was used to compare variables among the three phases. Post-tests were done as appropriate. Relationship between independent variables and Ca mass transfer was performed by Spearman correlation. General linear model (GLM) repeated measures were run to determine mean Ca mass transfer differences among the three d[Ca] over time, and to examine differences between phases and the interaction between factors. Statistical analysis was performed with GraphPad Prism 5.0 (Ca, USA) and SPSS 21.0 (SPSS Inc. one Chicago, IL). Significance was assigned at p values ​​< 0.05.

## Results

**Fig 1:**
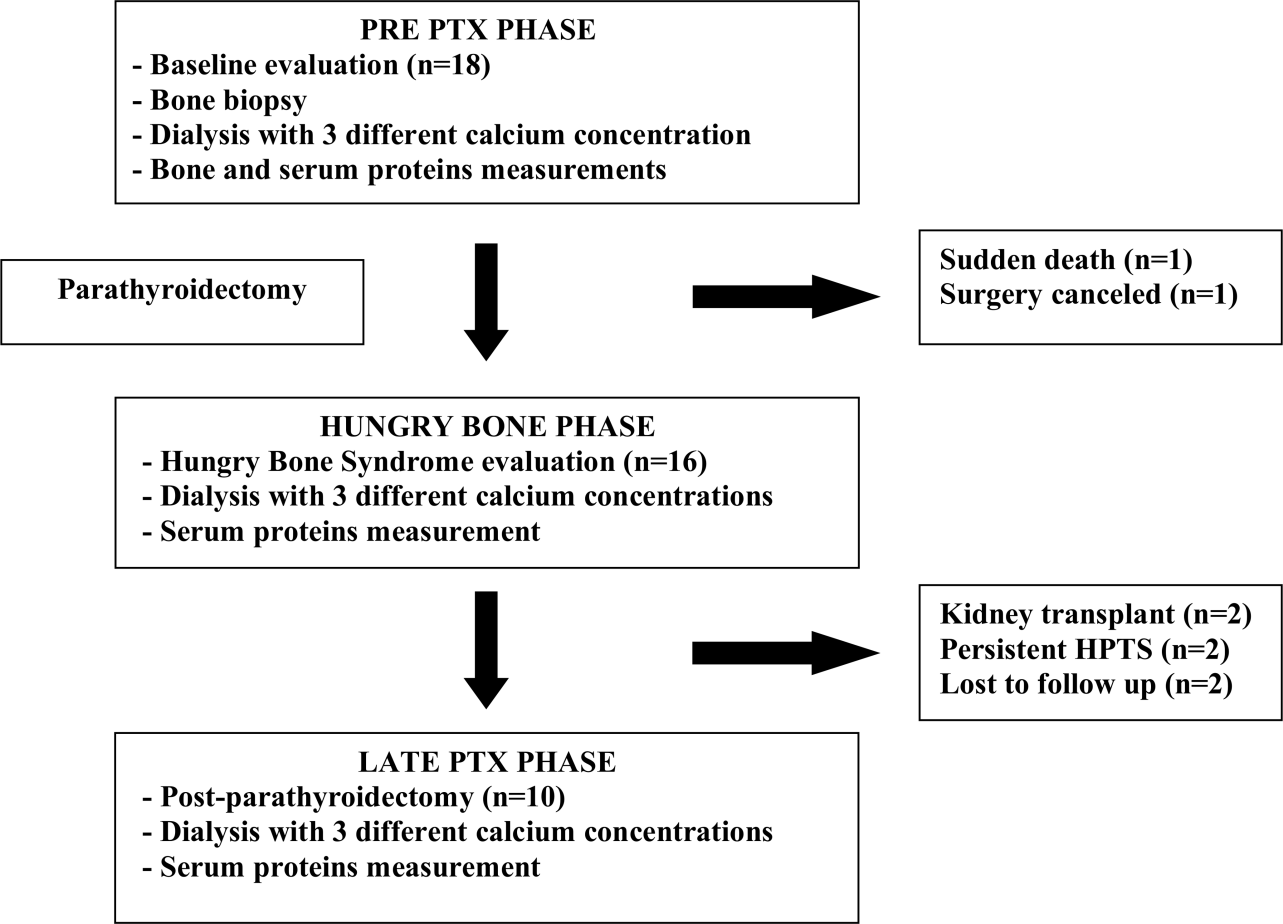
Schematic patients flow chart.

For study purposes, only the 10 patients who completed the 3 phases of the protocol were analyzed as described in Fig 1. Table 1 shows the characteristics of the study population. They were relatively young, 60% were men and most of them had been on dialysis for more than 4 years, and had clinical and laboratory manifestations of SHPT. Even though no patient had a history of bone fractures, more than a half complained of bone pain. During dialysis sessions, there were no serious adverse events.

**Table 1:** Patients characteristics.

Characteristics	Pre-PTX	Early post-PTX	Late post-PTX	Reference Values
**Male sex (n;%)**	6 (60%)	-	-	-
**Age (years)**	48.1 ± 11.0	-	-	-
**Dialysis vintage (Mo)**	57.6 ± 16.8	-	-	-
**Hypertension (n;%)**	8 (80%)	-	-	-
**Diabetes (n)**	0	-	-	-
**Bone pain (n;%)**	6 (60%)	-	-	-
**Fracture (n;%)**	0	-	-	-
**PTH**	1896 ± 648	50 ± 43	182 ± 110	12–65 pg/ml
**Total Ca**	2.4 ± 0.08	2.22 ± 0.38	1.95 ± 0.3 ***	2.15–2.55 mmol/L
**Ionized Ca**	1.23 ± 0.05	1.19 ± 0.19	1.04 ± 0.15	1.15–1.32 mmol/L
**Phosphate**	2.0 ± 0.35*	1.13 ± 0.35	1.7 ± 0.65	0.87–1.45 mmol/L
**Alkaline Phosphatase**	150 (102; 317)	226 (157; 503)	62 (46; 98) *	40–129 U/L
**25 vitamin D**	30.0 ± 13.1	27.1 ± 8.0	33.7 ± 11.4	30–100 ng/ml
**Bicarbonate**	21.0 ± 2.4	22.1 ± 3.5	18.9 ± 2.3***	23–27 mmol/L
**Albumin**	4.2 ± 0.3	4.1 ± 0.4	4.3 ± 0.2	3.4–4.8 g/dl
**Calcitriol use (n;%)**	2 (20)*	10 (100)	2 (20)*	-
**Sevelamer use (n;%)**	8 (80)*	0 (0)	5 (50) *	-
**CaCO**_**3**_ **use (n;%)**	1 (10)*	9 (90) **	6 (60) *^;^ ***	-

HAS = hypertension, DM = diabetes mellitus, PTH = parathormone, CaCO3 = calcium carbonate. Results expressed as mean ± standard deviation or median (25;75%), as appropriate.

*p<0.05 versus Early post-PTX

** p<0.05 versus Late post-PTX

*** p<0.05 versus Pre PTX

### a. Ca mass transfer during dialysis

**Fig 2:**
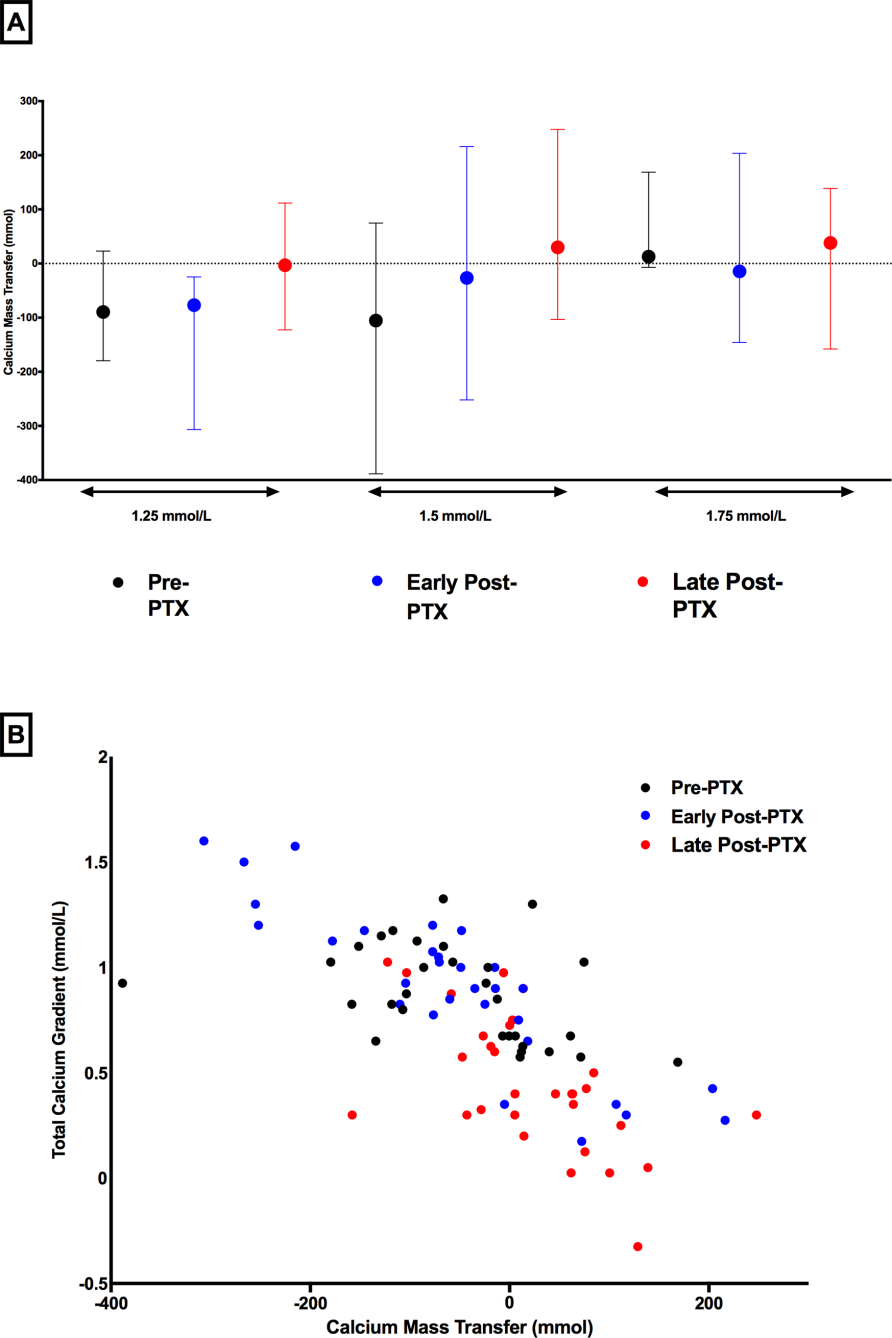
Plot of Ca mass transfer according to d[Ca] 1.25, 1.5 and 1.75 mmol/L in each phase of the study: Pre PTX (black), Early post-PTX (blue) and Late post-PTX (red). Markers and lines represent median and minimal and maximum values of Ca mass transfer (A). Relationship between Ca gradient (using total Ca) and Ca mass transfer in each phase of the study. Markers represent results from Pre PTX (black), Early post-PTX (blue) and Late post-PTX phases (red) (B).

**Fig 3:**
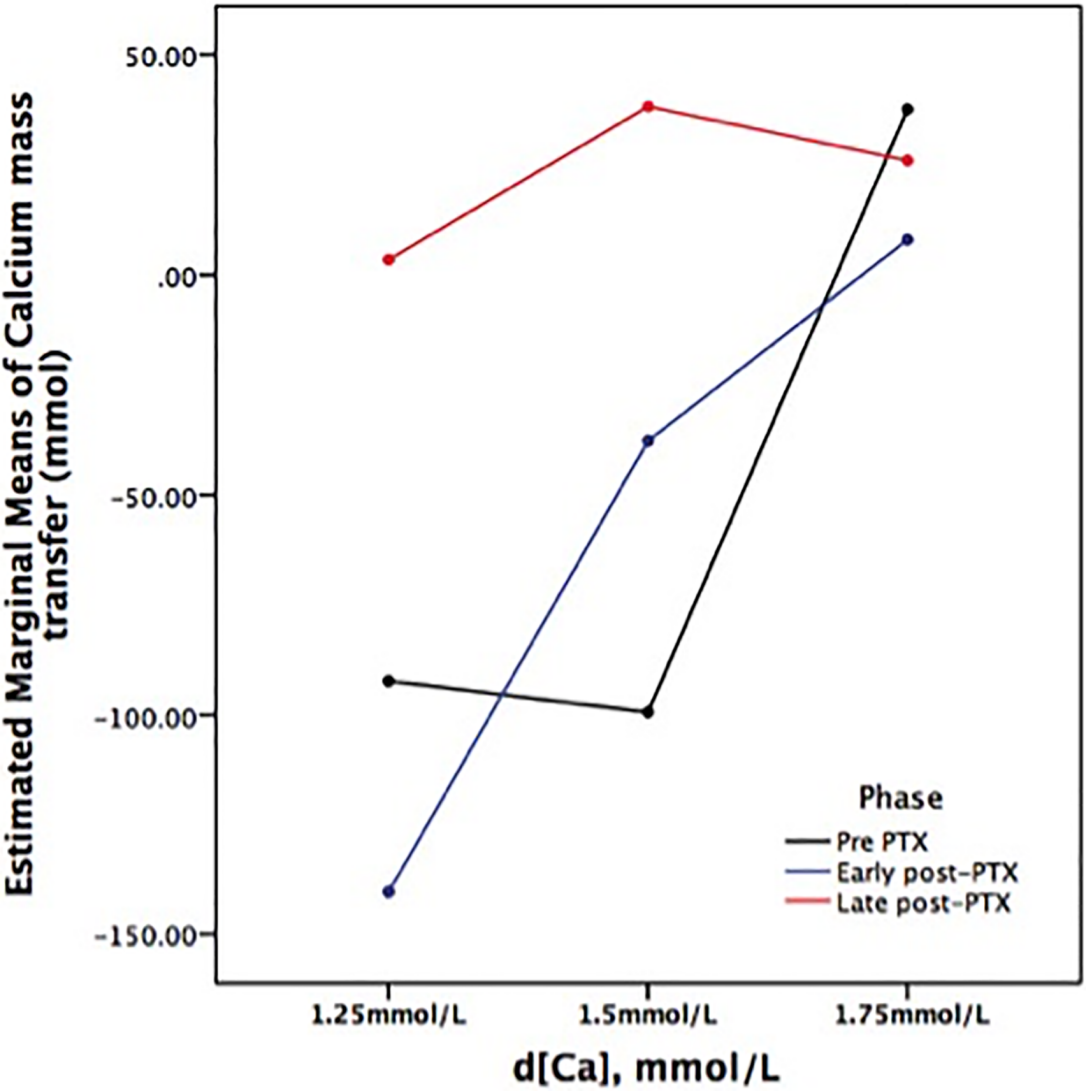
Plot generated from repeated measures General Linear Model, showing the entire group in all phases (90 dialysis sessions), according to dialysate calcium content–d[Ca] and study phases (Pre PTX in black, Early post-PTX in blue and Late post-PTX in red).

Based on changes on total and ionized calcium from pre to post hemodialysis (ΔtCa and on the ΔiCa, respectively), we would expect a neutral or negative Ca balance by using d[Ca] of 1.25 mmol/l in the Pre PTX and in the Early post-PTX phases, and positive in all other situations tested in our protocol. However, there was a wide variation on Ca mass transfer among patients (Fig 2A). The amplitude of variation occurred even at similar d[Ca] and changed through the study phases, confirming the hypothesis that is very difficult to predict the Ca mass transfer based exclusively on the d[Ca]. Table 2 illustrates median and 25th, 75th percentile of Ca mass transfer according to d[Ca] and each study phase. Correlations of Ca mass transfer with Ca gradient (tCa and iCa) (Fig 2B and S1 Fig, respectively) confirm that different Ca mass transfer could be seen in a similar Ca gradient. Ca mass transfer was mostly negative using d[Ca] 1.25 mmol/l and positive using d[Ca] 1.75mmol/l. Looking at Ca mass transfer according to bone remodeling status and regardless the d[Ca], we found negative median values in Pre PTX and early post-PTX (-40.25 and -54.5 mmol, respectively) and slightly positive in late post-PTX (9.75 mmol; p<0.05 versus Pre PTX and early post-PTX). The influence of bone remodeling on Ca mass transfer was established by GLM analysis, as during late post-PTX phase, there was a significantly higher Ca mass transfer as compared to Pre PTX and early post-PTX phases (Fig 3).

**Table 2:** Calcium gradients, balances and variations.

	d[Ca] 1.25 mmol/L			d[Ca] 1.5 mmol/L			d[Ca] 1.75 mmol/L					
	Pre PTX	Early post-PTX	Late post-PTX	Pre PTX	Early post-PTX	Late post-PTX	Pre PTX	Early post-PTX	Late post-PTX	P Among d[Ca]	P Among Phases	P Interaction
**ΔtCa (mmol/L)**	0.05 (-1.06, 0.14)	-0.11 (-0.38, 0.12)	0.31 (0.19, 0.49)	0.25 (0.14, 0.31)	0.38 (-0.08, 0.59)	0.60 (0.34, 0.66)	0.43 (0.25, 0.56)	0.21 (0.06, 0.48)	0.74 (0.59, 0.89)	0.0001 ^a,c^	0.001^#^^,^*	0.082
**ΔiCa (mmol/L)**	-0.21 (-0.47, 0.08)	-0.08 (-0.75, 0.23)	0.33 (-0.10, 0.71)	0.32 (0.02, 0.50)	0.30 (-0.14, 0.77)	0.71 (0.35, 1.07)	0.80 (0.22, 0.94)	0.47 (-0.08, 1.17)	1.26 (1.07, 1.58)	0.0001^a,b,c^	0.003^#^^,^*	0.640
**ΔPTH (pg/ml)**	126 (-456, 2091)	5 (-26, 32.2)	3 (-49, 76)	-632 (-1054, 5174)	-6 (-80, 14)	-34 (-109, 18)	-829 (-1992, 216)	17 (2, 58)	-93 (-230, 46)	0.062	0.482	0.052
**Ca grad tCa (mmol/L)**	-0.03 (-0.47, 0.31)	-0.27 (-1.48, 0.71)	-0.78 (-1.72, 0)	-1.01 (-1.71, -0.78)	-1.22 (-2.33, -0.01)	-1.78 (-2.84, -0.66)	-1.89 (-2.25, -1.69)	-1.81 (-3.55, -0.66)	-3.02 (-4.30, -2.12)	0.0001^a,b,c^	0.007^#^^,^*	0.365
**Ca grad iCa (mmol/L)**	1.11 (1.02, 1.20)	0.86 (0.82, 0.94)	0.61 (0.58, 0.68)	1.13 (0.84, 1.5)	0.86 (0.33, 1.14)	0.90 (0.40, 1.04)	0.70 (0.46, 0.90)	0.40 (0.25, 0.69)	0.30 (0.04, 0.34)	0.0001^a,b,c^	0.0001^#^*	0.007
**Ca mass transf. (mmol)**	-89.5 (-180, 23)	-76.8 (-307, -25)	-3 (-122, 112)	-106 (-389, 75)	-26.8 (-252, 216)	29.7 (-103, 248)	12.8 (-7, 169)	-14.5 (-146, 204)	38 (-158, 139)	0.0001^c^	0.040^#^^,^*	0.008
**UF (L)**	3.25 (1.8, 4.0)	4.0 (1.7, 4.5)	3.2 (2.0, 4.0)	3.2 (1.5, 4.0)	3.6 (1.2, 4.5)	2.7 (2.0, 4.5)	3.3 (1.6, 4.5)	3.8 (2.5, 4.0)	2.9 (0.8, 4.5)	0.834	0.223	0.904

Results expressed as mean ± SD or median (25;75%), as appropriate. ΔiCa, and ΔPTH = difference between post and pre dialysis values of iCa and PTH, respectively. Post test among d[Ca]: *a* p<0.05 1.25 vs. 1.5 mmol/L; *b* p<0.05 1.5 vs. 1.75 mmol/L; *c* 1.25 vs. 1.75 mmol/L. Post test among phases:

# p<0.05 Pre PTX vs. Late post-PTX phase

* p<0.05 Early post-PTX vs. Late post-PTX phase

Gathering all 90 dialysis sessions, we found that Ca mass transfer correlated with pre dialysis iCa (r = -0.52; p< 0.0001), tCa (r = -0.73; p< 0.0001), alkaline phosphatase (r = -0.40; p< 0.0001), serum GLU (r = -0.39; p< 0.0001), OC (r = -0.29; p = 0.006), tCa gradient (r = -0.66; p< 0.0001), ΔtCa (r = 0.55; p< 0.0001), iCa gradient (r = -0.69; p< 0.0001), and ΔiCa (r = 0.61; p< 0.0001).

### b. Ca gradient, iCa and PTH changes during dialysis

**Fig 4:**
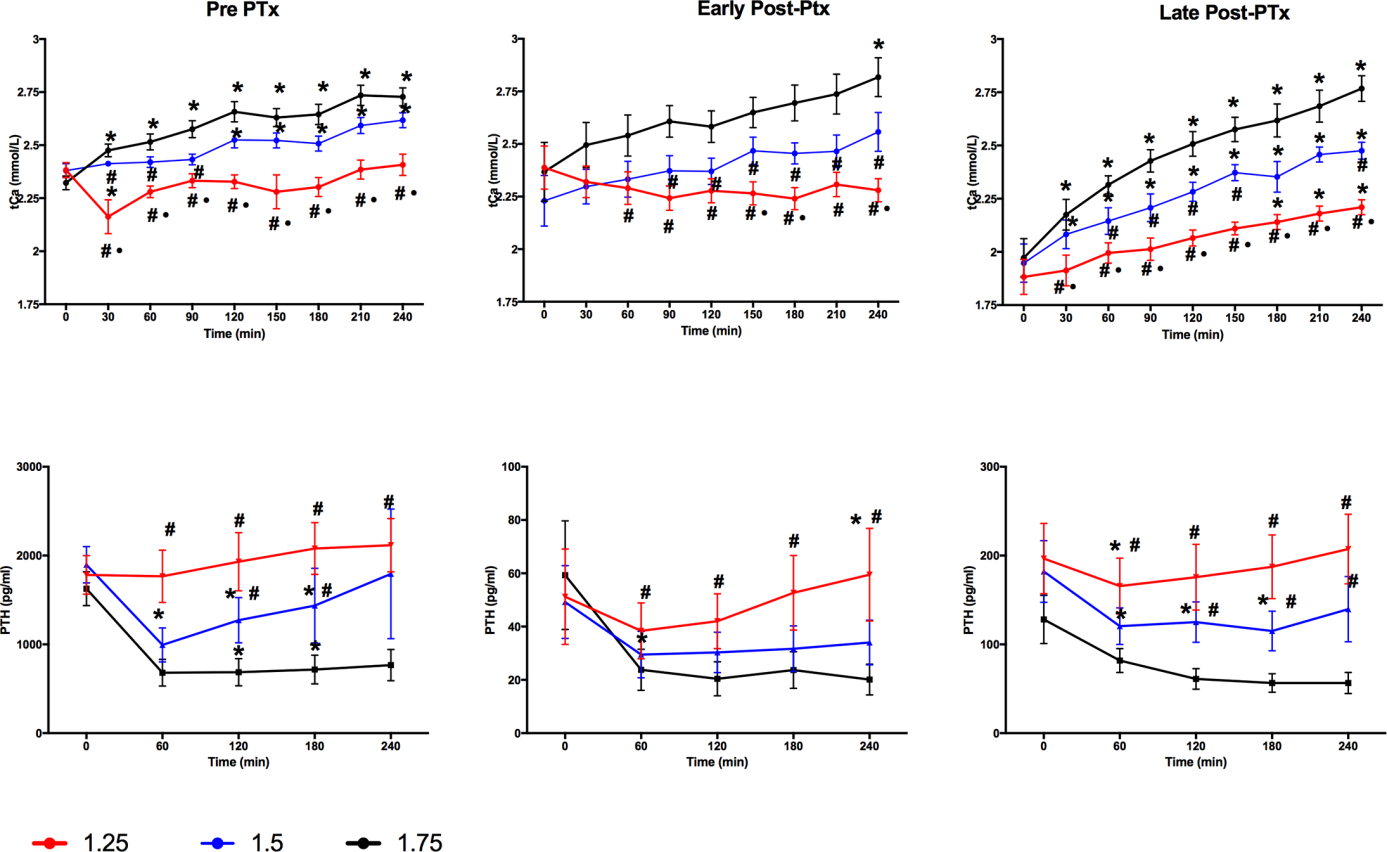
Calcium and PTH curves throughout the phases of study. Red line represents d[Ca] 1.25, blue line represents d[Ca] 2.5 and black line represents d[Ca] 1.75 mmol/l. Differences are marked when significant as follow: * for p<0.05 vs. time 0 (intra-group comparisons), # for p<0.05 vs. d[Ca] 1.75 mmol/l and for p<0.05 vs. d[Ca] 1.5 mmol/l (inter-group comparisons).

The variations in tCa, iCa and PTH from pre to post dialysis (ΔtCa, ΔiCa and ΔPTH, respectively) are shown in Table 2 and Fig 4 and S2 Fig. ΔtCa and ΔiCa were significantly different among all three d[Ca] studied. ΔtCa and ΔiCa were higher during the late post-PTX when compared to Pre PTX and early post-PTX phases. The same behavior was observed regarding Ca gradient. Curiously, hemodialysis promoted a decrease in serum tCa only with a d[Ca]1.25 mmol/l during Early post-PTX phase, whereas a decrease in iCa was seen only with a d[Ca]1.25 mmol/l during Pre PTX and Early post-PTX phases. The greatest variation of PTH was observed during Pre PTX, although not significant. Ultrafiltration volume was similar among all phases and all d[Ca].

### c. Serum protein analysis

Serum OC, as well as its fractions GLU and GLA, decreased only in the Late post-PTX phase, as seen in Table 3. Conversely, serum sclerostin increased during the Early post-PTX phase and persisted elevated in the Late post-PTX phase. No significant changes were observed in the serum concentration of any other protein.

**Table 3:** Serum proteins according to the phase.

Proteins			
	Pre PTX	Early post-PTX	Late post-PTX
OC	80.8 ± 24.4^#^	94.2 ± 22.5^#^	58.0 ± 20.3
GLA	245.5 (110.2; 321.9) ^#^	151.5 (82.1; 404.0) ^#^	11.84 (0.7; 104.3)
GLU	221.7 (103.6; 609.9) ^#^	159.5 (89.8; 435.8) ^#^	10.0 (4.2; 32.1)
SCL	0.79 ± 0.31*^,^^#^	1.06 ± 0.28	1.15 ± 0.23
Leptin	11.3 (8.5; 25.1)	13.2 (7.0; 41.0)	20.0 (10.2; 48.0)
FGF23	4.5 ± 1.9	3.1 ± 1.4	3.6 ± 3.2
DKK1	2.2 ± 0.9	2.1 ± 0.5	2.3 ± 0.5
OPG	1.5 ± 0.5	1.8 ± 0.5	1.9 ± 0.4

Serum proteins concentration are expressed in ng/ml. OC- osteocalcin, GLA- carboxylated osteocalcin, GLU- undercarboxylated osteocalcin, SCL- sclerostin, FGF23- fibroblast growth factor 23, DKK1- Dikkopf 1, OPG- osteoprotegerin. Results are expressed as mean ± SD or median (25;75%), as appropriate.

*p<0.05 versus Early post-PTX

^#^ p<0.05 versus Late post-PTX.

## Discussion

In the present study we evaluated the mass transfer of Ca from dialysis at different levels of d[Ca] during different states of bone remodeling. The results demonstrate that the blood Ca remains the most important factor in determining the Ca gradient, which in turn affects net Ca mass transfer. The Ca gradient is determined by the difference between serum Ca and d[Ca], and the large variation in the Ca in the same patient during each treatment will thus alter the gradient, and in turn the Ca mass transfer. Previous studies have shown that Ca gradient is more accurate when based on total Ca, since it includes the complexed and diffusible fraction [15, 18]. Interestingly, the Ca gradient to mass transfer relationship (Fig 2B and S1 Fig) was similar whether ionized Ca or total Ca was used. This should facilitate the ability of clinicians to individualize the approach to patients to optimize d[Ca] to avoid excess Ca mass transfer.

The state of bone remodeling also plays a role in Ca mass transfer although the relationship was far more complicated. This might be possible either because it influences serum Ca or directly as it controls the Ca available to be dialyzed. We hypothesized that the skeleton has a surface compartment that “buffers” the ionized Ca and provides acute buffering during dialysis that would significantly impact the Ca mass transfer. However, we did not see large differences in the three phases of bone remodeling in Ca mass transfer (Fig 2A). Indeed, we were expecting a positive Ca mass transfer during the early post-PTX phase due to a continuous influx of Ca into bone due to rapid bone formation/mineralization rather than bone resorption, which was not observed. In 1994, Kurz et al [21] performed double radiolabeled Ca and found that the acute Ca accretion (bone uptake) was greatest in patients with high turnover bone disease compared to either mixed uremic osteodystrophy or low turnover bone when studied on a non-dialysis day. However, the net Ca retention, the fraction of the intravenously administered Ca retained 4 weeks after injection was not different among the patients with the different bone histology groups. The latter may imply that dialysis has more of an impact on net Ca mass transfer than the underlying bone histology and therefore the ability to detect acute bone buffering may be limited during dialysis. Our findings are consistent with those of Sigrist et al. [11] in that the greatest predictor of Ca mass transfer during a dialysis session was the Ca gradient. However, based on our results, the role of bone, even if indirect, should not be neglected. Supporting our hypothesis, Talmage et al. [22] have already demonstrated in parathyroidectomized rats that bone was able to continuously supply calcium to the extracellular fluid at high rates during calcium-free peritoneal dialysis.

The baseline tCa and iCa levels were similar in the Pre-PTX and early post-PTX phases of the study. We hypothesize this may be explained by the different doses of Ca salts and calcitriol in each of the phases. In contrast, in the Late post-PTX phase when blood levels were likely more stable requiring less medication, the total and ionized Ca levels were nearly 0.45 and 0.25 mmol/L lower, respectively. The determinants of serum Ca level include not only Ca intake, but also the ability of bone to regulate Ca levels. While bone remodeling may take weeks to months to change in response to PTH, there is a buffering capacity of bone due to surface proteins such as OC. In the present study, in the Late post-PTX, we found much lower levels of OC (total, carboxylated, and uncarboxylated) and lower PTH. PTH may in part, regulate the abundance of the surface proteins. Talmage et al. [17] have suggested that PTH, besides stimulation of bone osteoclast resorption, could act by either changing the conformation, the amount of OC, or by removing interfering substances [13]. The importance of these surface proteins in taking up Ca was demonstrated in the study by Kurz et al. detailed above [21]. Authors found that the correlation of bone Ca accretion rate was tightly correlated with OC and alkaline phosphatase and less so with PTH.

Similarly, our group [8] has previously shown that the cross sectional analyses of Ca mass transfer demonstrated wide variability. However, multivariate analyses suggested that both the OC levels and the PTH levels could explain, at least partially, the variability. In the present study, each patient was studied with different d[Ca] and different levels of bone remodeling and the results suggest that the important role of bone may have more to do with the blood Ca levels rather than due to acute fluxes with dialysis. In patients in Late post-PTX, there is a lower serum Ca level and a decrease in bone buffering capacity due to reduced bone surface proteins. This may explain why there was less variability in Ca mass transfer regardless of the d[Ca ]. Thus, both the long term bone remodeling and the acute bone buffering may be limited post PTX resulting in lower ambient Ca levels.

The present study has some limitations. First, the sample size was relatively small. However, the study design could partially overcome this problem, as patients were their own controls. Also, we enrolled only patients with SHPT and thus our results may not apply to patients with mild hyperparathyroid bone disease. In addition, we used standard thrice-weekly hemodialysis and our results may not be applicable to other more intensive dialysis regimens, where the ideal d[Ca] is still debatable [23, 24]. We also did not enroll any diabetic patients, probably due to the fact that these patients commonly have low, rather than high turnover bone disease and rarely require PTX. Also, although both iCa and tCa correlated with Ca mass transfer, we are aware of the existence of the different pools of calcium in blood and the fact that Ca is diffusible and can be complexed with phosphate and citrate, which make difficult in obtaining accurate measurement in the spent dialysate. Our study has also some strength as provided new insights in the Ca mass transfer process during hemodialysis, describing not only the role of Ca gradient but also showing the inter- and intra-patient variation according to bone turnover status.

In summary, our results showed that Ca mass transfer during hemodialysis is highly variable but is more dependent on the given ionized (or total) Ca during the treatment. The latter, in turn may depend on the bone’s ability to regulate Ca levels. We believe our results suggest that d[Ca] should be determined based on the patient’s serum Ca level and net intake of dietary and Ca containing phosphate binders. In patients receiving exogenous Ca from phosphate binders, the goal would be a negative Ca mass transfer in order to maintain more neutral overall balance. This can only be accomplished if the Ca dialysate is less than the patient’s serum levels. Fortunately, our results were similar with total Ca and ionized Ca making this approach more suitable in clinical practice. While not tested in this study, our findings also suggest that in patients receiving a calcimimetic, with lower serum Ca, the use of higher (than serum) Ca dialysate may lead to greater net Ca mass transfer. Taken together, our study suggests that the standard practice of choosing only one d[Ca] concentration per dialysis unit, a “one size fits all” approach, does not work. Practitioners prescribe ultrafiltration, sodium, and bicarbonate based on an individual patient’s weight and laboratory values, and we believe Ca should be similarly managed based on pre dialysis Ca, d[Ca] and bone remodeling status.

## Supporting information

S1 Fig Relationship between Ca gradient (using ionized Ca) and Ca mass transfer in each phase of the study. Markers represent results from Pre PTX (black), Early post-PTX (blue) and Late post-PTX phases (red) (B).
(TIFF)

S2 Fig Ionized Calcium and PTH curves throughout the phases of study. Red line represents d[Ca] 1.25, blue line represents d[Ca] 2.5 and black line represents d[Ca] 1.75 mmol/l. Differences are marked when significant as follow: * for p<0.05 vs. time 0 (intra-group comparisons), # for p<0.05 vs. d[Ca] 1.75 mmol/l and for p<0.05 vs. d[Ca] 1.5 mmol/l (inter-group comparisons).
(TIFF)
