# Supplementary figures and images for: A prospective study of the influence of the skeleton on calcium mass transfer during hemodialysis
Source: PLoS One. 2018 Jul 30;13(7):e0198946. doi: 10.1371/journal.pone.0198946 (PMC6066217; doi:10.1371/journal.pone.0198946)

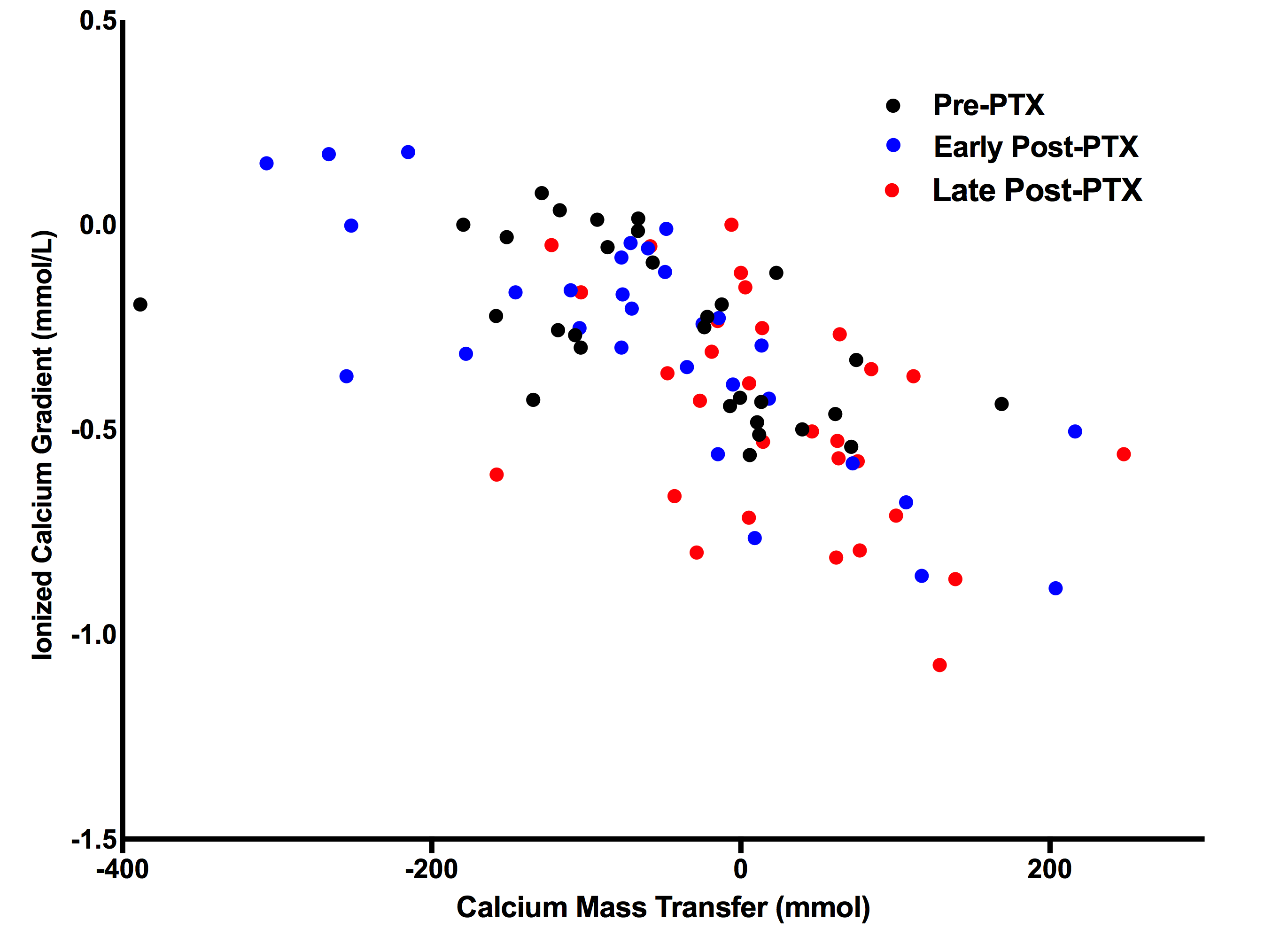

Supplement: S1 Fig — Markers represent results from Pre PTX (black), Early post-PTX (blue) and Late post-PTX phases (red) (B). (TIFF) [file pone.0198946.s001.tiff]

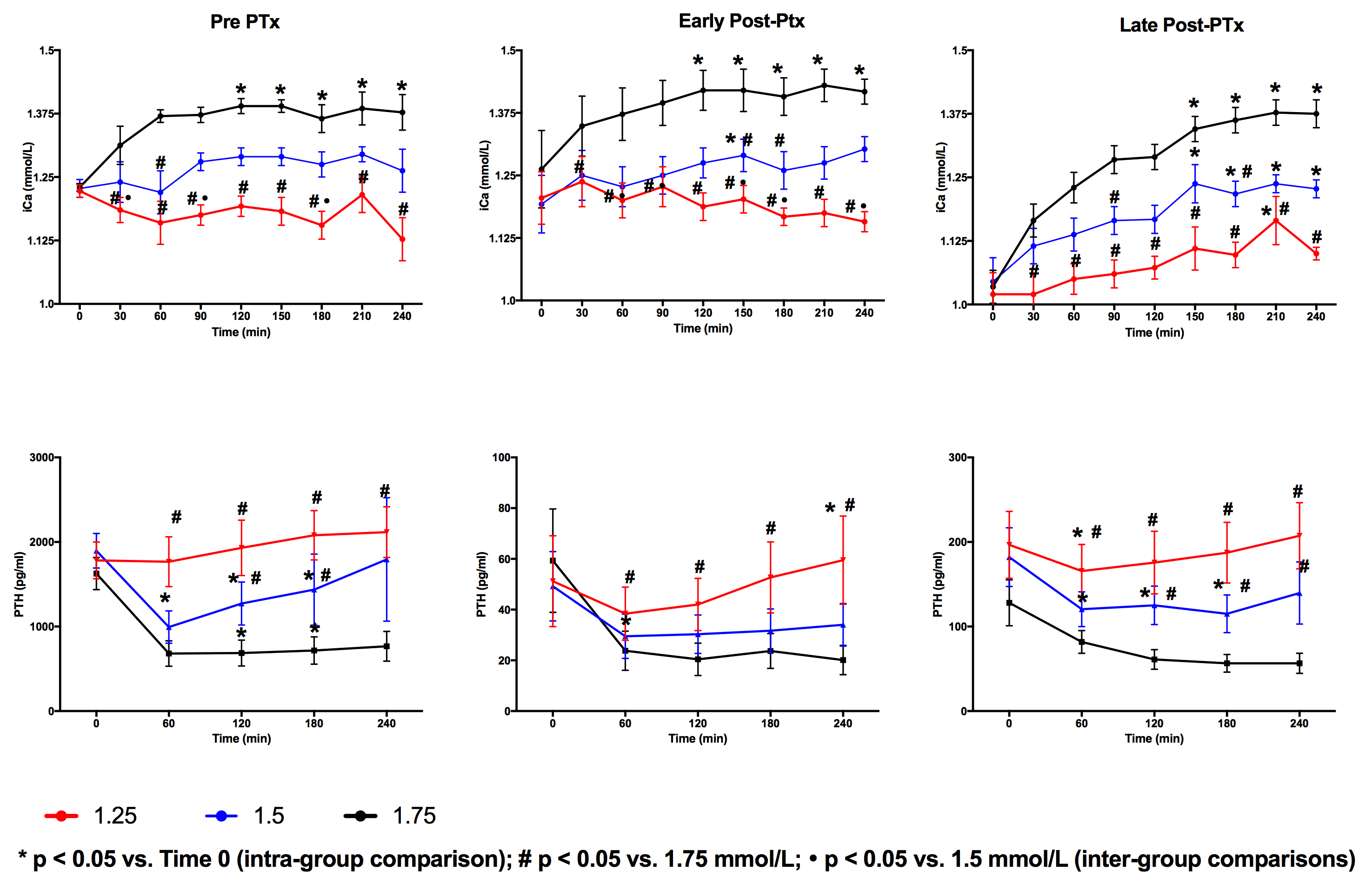

Supplement: S2 Fig — Red line represents d[Ca] 1.25, blue line represents d[Ca] 2.5 and black line represents d[Ca] 1.75 mmol/l. Differences are marked when significant as follow: * for p<0.05 vs. time 0 (intra-group comparisons), # for p<0.05 vs. d[Ca] 1.75 mmol/l and for p<0.05 vs. d[Ca] 1.5 mmol/l (inter-group comparisons). (TIFF) [file pone.0198946.s002.tiff]
